# Comparison of RNA synthesis initiation properties of non-segmented negative strand RNA virus polymerases

**DOI:** 10.1371/journal.ppat.1010151

**Published:** 2021-12-16

**Authors:** Afzaal M. Shareef, Barbara Ludeke, Paul Jordan, Jerome Deval, Rachel Fearns

**Affiliations:** 1 Department of Microbiology, Boston University School of Medicine, Boston, Massachusetts, United States of America; 2 Janssen Biopharma, South San Francisco, California, United States of America; Washington University in Saint Louis School of Medicine, UNITED STATES

## Abstract

It is generally thought that the promoters of non-segmented, negative strand RNA viruses (nsNSVs) direct the polymerase to initiate RNA synthesis exclusively opposite the 3´ terminal nucleotide of the genome RNA by a *de novo* (primer independent) initiation mechanism. However, recent studies have revealed that there is diversity between different nsNSVs with pneumovirus promoters directing the polymerase to initiate at positions 1 and 3 of the genome, and ebolavirus polymerases being able to initiate at position 2 on the template. Studies with other RNA viruses have shown that polymerases that engage in *de novo* initiation opposite position 1 typically have structural features to stabilize the initiation complex and ensure efficient and accurate initiation. This raised the question of whether different nsNSV polymerases have evolved fundamentally different structural properties to facilitate initiation at different sites on their promoters. Here we examined the functional properties of polymerases of respiratory syncytial virus (RSV), a pneumovirus, human parainfluenza virus type 3 (PIV-3), a paramyxovirus, and Marburg virus (MARV), a filovirus, both on their cognate promoters and on promoters of other viruses. We found that in contrast to the RSV polymerase, which initiated at positions 1 and 3 of its promoter, the PIV-3 and MARV polymerases initiated exclusively at position 1 on their cognate promoters. However, all three polymerases could recognize and initiate from heterologous promoters, with the promoter sequence playing a key role in determining initiation site selection. In addition to examining *de novo* initiation, we also compared the ability of the RSV and PIV-3 polymerases to engage in back-priming, an activity in which the promoter template is folded into a secondary structure and nucleotides are added to the template 3´ end. This analysis showed that whereas the RSV polymerase was promiscuous in back-priming activity, the PIV-3 polymerase generated barely detectable levels of back-primed product, irrespective of promoter template sequence. Overall, this study shows that the polymerases from these three nsNSV families are fundamentally similar in their initiation properties, but have differences in their abilities to engage in back-priming.

## Author summary

The non-segmented negative strand RNA viruses are a large group of viruses that includes a number of significant human pathogens. Their viral genome is transcribed and replicated by a virally-encoded polymerase, which is considered to be a good target for intervention with antiviral drugs. Defining the similarities and differences between the polymerases of different viruses could be helpful for developing inhibitors with potency against a broad spectrum of related viruses. In this study, we examined the RNA synthesis initiation properties of polymerases of viruses from three different non-segmented negative strand RNA virus families and showed that they are fundamentally similar in the ways that they can initiate RNA synthesis. However, we also found that polymerases from two different virus families differed significantly in their ability to perform another activity, referred to as back-priming, which suggests that there might be structural differences between them. Thus, this study identifies similarities and differences between the polymerases in this group of viruses.

## Introduction

The order *Mononegavirales*, otherwise known as the non-segmented, negative strand RNA viruses (nsNSVs), is a large and diverse group of viruses that are united by having a genome consisting of a single strand of RNA that is transcribed to produce a series of mRNAs, and replicated via a positive-sense antigenome intermediate to produce new RNA genomes [1]. They also all encode a multi-functional polymerase to perform transcription and genome replication [2,3]. The nsNSVs encompass several viral families and the order includes several significant pathogens, such as vesicular stomatitis virus (VSV) and rabies virus (family *Rhabdoviridae*), Zaire ebolavirus (EBOV) and Marburg (MARV) viruses (family *Filoviridae*), human parainfluenza virus type 3 (PIV-3) (family *Paramyxoviridae*), and respiratory syncytial virus (RSV) and human metapneumovirus (HMPV) (family *Pneumoviridae*). Because the nsNSV polymerases are enzymes that are essential for viral replication and have properties that are distinct from cellular enzymes, they are good targets for intervention with small molecule inhibitors [4,5]. Although the nsNSVs share many mechanistic aspects of transcription and genome replication, and their polymerases have a high degree of conservation, there are some differences between them [3]. A greater understanding of the similarities and differences between these polymerases and their functional properties can help us to appreciate the biology of the nsNSV order and could potentially be leveraged in the development of broad-spectrum inhibitors.

All nsNSV genomes contain a single promoter element located at the genome 3´ end called the *leader* (*le*) promoter, which engages the polymerase to initiate both transcription and replication (note that throughout this paper, the promoter and other *cis*-acting element names are italicized, whereas sequence complementary to the promoter is not). The antigenome RNA replication intermediate also has a promoter at its 3´ end, called the *trailer* (*tr*) promoter [6], which directs initiation of genome RNA synthesis. While there is evidence that the nsNSV polymerase recognizes the viral genome and antigenome templates as nucleoprotein (N or NP) -RNA complexes, the initiation complex is almost certainly assembled on naked RNA from which N or NP protein has been locally dissociated. This is evident from the fact that the nsNSV polymerase structures that are available show that there is insufficient space in the template channel to accommodate an N-RNA structure [7–13], indicating that RNA synthesis initiation occurs after the RNA has dissociated from N and entered the polymerase active site. In addition, the RSV and VSV *le* promoters each contain several adjacent specific nucleotides, which would not all be exposed in N-RNA [14–18]. The *le* and *tr* promoters typically have a high degree of similarity within each virus but differ in sequence between different viruses. They also differ in the locations of initiation sites within the promoter [19]. Previously, we performed studies to determine the mechanism of RSV transcription and replication initiation. Analysis of RNA from RSV-infected cells and biochemical studies of RSV polymerase activity on a naked RNA promoter template revealed that the RSV *le* and *tr* promoters each have two initiation sites, at positions 1U and 3C [20–22]. RNA initiated at 1U can be encapsidated and extended, consistent with this being the replication initiation site. RNA initiated at position 3C is released after ~25 nt [22,23]. In the case of the *le* promoter at the 3´ end of the genome, the short ~25 nt transcript represents the first step of transcription and following its release the polymerase can then go on to transcribe the RSV genes [22,23]. In the case of the *tr* promoter, the function of the short RNA is not known, but it might have a role in subverting cellular stress granule formation [24]. Initiation at the 1U and 3C sites of the RSV promoters is precisely controlled (i.e. the polymerase does not initiate at other sites on the promoters) [22,25], but the mechanisms underlying this control are not completely understood. Studies of the HMPV polymerase have shown that it initiates at positions 1U and 3C on its *le* promoter, indicating that HMPV has a similar mechanism of transcription initiation as RSV [13]. Ebolavirus polymerases can also initiate at an internal site [26]. Specifically, ebolavirus genome and antigenome RNAs were found to be variable at their 3´ ends, with some RNAs having an additional 3´ A or G residue that was not reflected at the 5´ end of the encoded RNA. Experiments using an EBOV minigenome showed that the polymerase could initiate RNA replication at the motif 3´ CCUGU, regardless of the presence or absence of the additional 3´ A or G residue [26]. In contrast to the RSV, HMPV and EBOV polymerases, other nsNSV polymerases do not appear to initiate at an internal site on the promoter [17,27]. In the case of VSV polymerase, even addition of one to three nucleotides to the 3´ end of the promoter inhibited its activity [17,27]. This indicates that the VSV polymerase has structural features that prevent the 3´ end of the template from overshooting the active site, whereas the RSV, HMPV and EBOV polymerases allow for more flexibility in template positioning.

In addition to using the *tr* promoter as a template for *de novo* RNA synthesis, *in vitro* studies of the RSV polymerase showed that it also engaged in a back-priming activity in which the RNA template was folded into a secondary structure and the 3´ end extended in a templated fashion to add the sequence GUC (and a smaller proportion of longer additions) [21]. Analysis of the 3´ end of antigenome-sense RNA isolated from RSV-infected cells showed that approximately one-third of these RNAs contained 1 to 3 nucleotides of the GUC sequence, indicating that *tr* promoter back-priming occurs in infected cells and is not simply an artifact of the *in vitro* assay [21]. Back-priming activity was also detected for the HMPV polymerase [13] and analysis of RNA from ebolavirus-infected cells suggested that the additional nucleotide present at the 3´ ends of the replicative RNAs could be added by a back-priming mechanism [26]. A back-priming activity has also been reported as an essential step of the genome replication mechanism of Borna disease virus [28]. In Borna disease virus, back-priming activity compensates for a viral endonuclease activity that renders the viral replicative RNAs free of a 5´ triphosphate moiety. The function of back-priming in the pneumoviruses and ebolaviruses is not well understood. In the case of EBOV, the additional nucleotide causes a slight increase in RNA synthesis initiation efficiency [26]. In the case of RSV, the GUC addition dampens promoter activity from a naked RNA template, and we have speculated that back-priming might represent a mechanism to regulate *tr* promoter activity under conditions in which N protein is limiting [21]. However, to date, it has not been possible to devise a strategy to inhibit back-priming in the context of ebolavirus or pneumovirus infection, without also inhibiting RNA synthesis initiation, and so its role during infection remains enigmatic. Any additional information that could be gained regarding back-priming could shed light on its significance. Back-priming activities have not been described for other nsNSV promoters, but they are not always easy to detect. In *in vitro* assays, the ability to detect the activity depends on the length of the template sequence and the identity of the radionucleotide used as the tracer. Evidence of the activity in RSV- and EBOV-infected cells depended on detailed analysis of relatively rare viral RNAs. Therefore, it is not clear if back-priming is an activity that is shared amongst many nsNSVs or is restricted to a subset of viruses.

A polymerase feature that might differ between nsNSVs and affect initiation of RNA synthesis and back-priming is a priming loop. This is a feature that is typically found in RNA dependent RNA polymerases that initiate RNA synthesis by a *de novo* (rather than primer-dependent) initiation mechanism [29]. The priming loop can help buttress the 3´ end of the template [30,31]. In addition, priming loops contain a ring-based or aromatic priming residue that can form base-stacking interactions with the initiating NTPs to help stabilize them in the active site for initiation [32–38]. In the case of influenza virus, the polymerase initiates opposite the 3´ terminal nucleotide to begin cRNA synthesis, but at position 4 to initiate vRNA synthesis (by a prime-realign mechanism) [39,40]. Correlating with this, the influenza virus polymerase priming loop adopts different conformations depending on which promoter it is associated with [37,40,41]. The fact that some nsNSV polymerases can initiate internally and engage in back-priming whereas others initiate exclusively at the 3´ end of the promoter could suggest that they might have different priming loop properties, with those polymerases that initiate internally and/or engage in back-priming having a very flexible priming loop (or no priming loop) and those that initiate exclusively at the 3´ end having a more fixed priming loop structure in the pre-initiation conformation.

To explore the similarities and differences between nsNSV polymerases, we expressed and purified RSV, PIV-3, and MARV polymerase complexes, to have representation from three different families, and examined their properties both on their cognate promoters, and promoters from other viruses. The results obtained showed that although different polymerases have different initiation preferences, all polymerases tested could efficiently initiate either at the 3´ terminal nucleotide of a template or an internal site, indicating that for these three viruses, the promoter sequence is a major factor determining which initiation site is utilized. However, there was a clear distinction between a polymerase that could engage in back-priming versus another that could not, indicating that this property is not shared by all nsNSVs.

## Results

### The RSV and PIV-3 polymerases differ in initiation properties on their respective promoters

**Fig 1:**
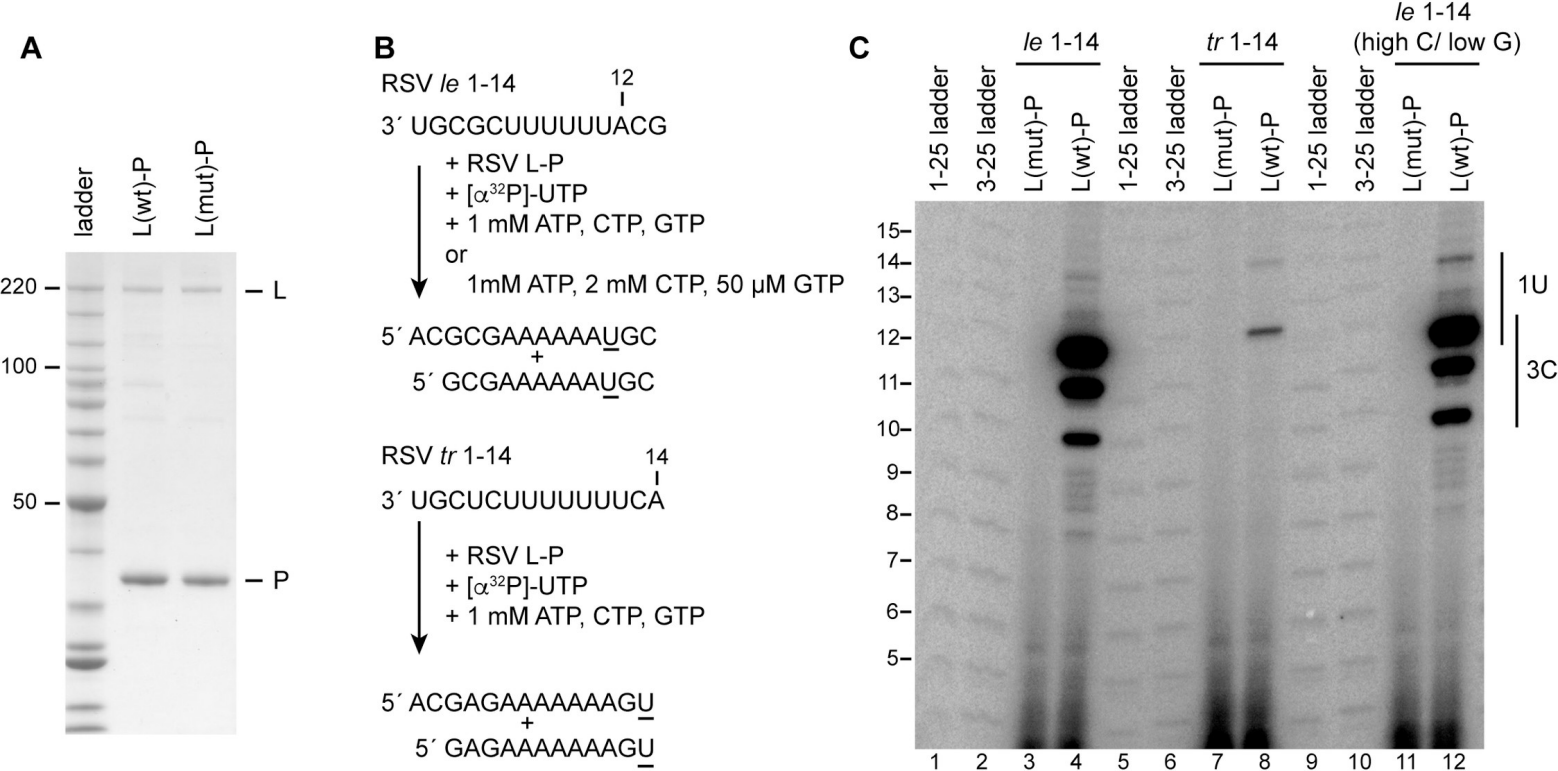
The RSV polymerase initiates at positions 1U and 3C of the RSV *le* and *tr* promoters. (A) SDS-PAGE analysis of wt and variant RSV L-P preparations detected by colloidal Coomassie blue staining. The variant L protein contains a substitution in the active site of the RNA polymerization domain. The first lane contains a Benchmark ladder. (B) Schematic diagram illustrating the RSV *le* 1–14 and *tr* 1–14 templates, the NTP concentrations used, and the full-length products that could be generated from the 1U and 3C initiation sites. The underlined residues indicate the sites of radiolabel incorporation. (C) RNA synthesis products generated by the wt and variant polymerase, analyzed by denaturing polyacrylamide gel electrophoresis. The 1–25 and 3–25 ladders were generated by alkali hydrolysis of ^32^P end-labeled RNAs representing the full-length complementary products generated from positions 1U and 3C of an RSV *tr* 1–25 template. Note that the ladders in lanes 1 and 2 were at the edge of the gel and migrated slightly more slowly than the ladders in lanes 5, 6, 9 and 10; the ladder size annotation is aligned to the internal ladder lanes.

As noted in the Introduction section, our previous studies have shown that the RSV polymerase initiates at positions 1U and 3C on both the *le* and *tr* promoters [20,22]. The results from a representative *in vitro* assay are shown in Fig 1. In this experiment, purified RSV L-P complexes of either wild type sequence, L(wt)-P, or with a D-to-A substitution in the GDN motif of the active site of the RNA dependent RNA polymerase domain, L(mut)-P (Fig 1A) were incubated with a 14 nt RNA oligonucleotide template, consisting of either RSV *le* or *tr* promoter sequence, in the presence of 1 mM each ATP, CTP and GTP, with a low concentration of [α-^32^P]-UTP included as a radioactive tracer (Fig 1B). In the case of the *tr* promoter, the first UTP incorporation site is at the 5´ end of the template at position 14. RNA synthesis reactions using this template yielded products of 12 and 14 nt in length, representing initiation at positions 3C and 1U, respectively, with initiation at position 3 being dominant (Fig 1C, lane 8). In the case of the *le* promoter template, the first UTP incorporation site is at position 12. Therefore, initiation at position 3C would yield radiolabeled products of 10–12 nt in length, whereas initiation at position 1U would yield radiolabeled products ranging from 12–14 nt. Analysis of RNAs produced from the RSV *le* 1–14 promoter showed that under equimolar ATP, CTP, and GTP conditions, products of 10–12 could be readily detected, consistent with initiation at position 3, but longer products from initiation at position 1 were much fainter (Fig 1C, lane 4). It should be noted that the levels of products from the *le* and *tr* promoters cannot be directly compared; all the polymerases examined in this paper were relatively inefficient at incorporating a (low concentration) radiolabeled nucleotide if this incorporation site corresponded to the 5´ end of the template. Thus, the signal from the *tr* promoter was much weaker than from the *le* promoter. When *le* promoter reactions were performed with biased NTP concentrations of 1 mM ATP, 2 mM CTP and 50 μM GTP, to increase efficiency of initiation from position 1, the 14 nt band could be detected more readily than in the unbiased NTP conditions, although the 10–12 nt bands were still dominant (Fig 1C, compare lanes 4 and 12). These findings are consistent with the RSV promoters directing the RSV polymerase to initiate at positions 1U and 3C, with initiation at 3C being highly dominant, especially in the case of the *le* promoter. These results mirror findings from analysis of RNA from RSV-infected cells [22].

**Fig 2:**
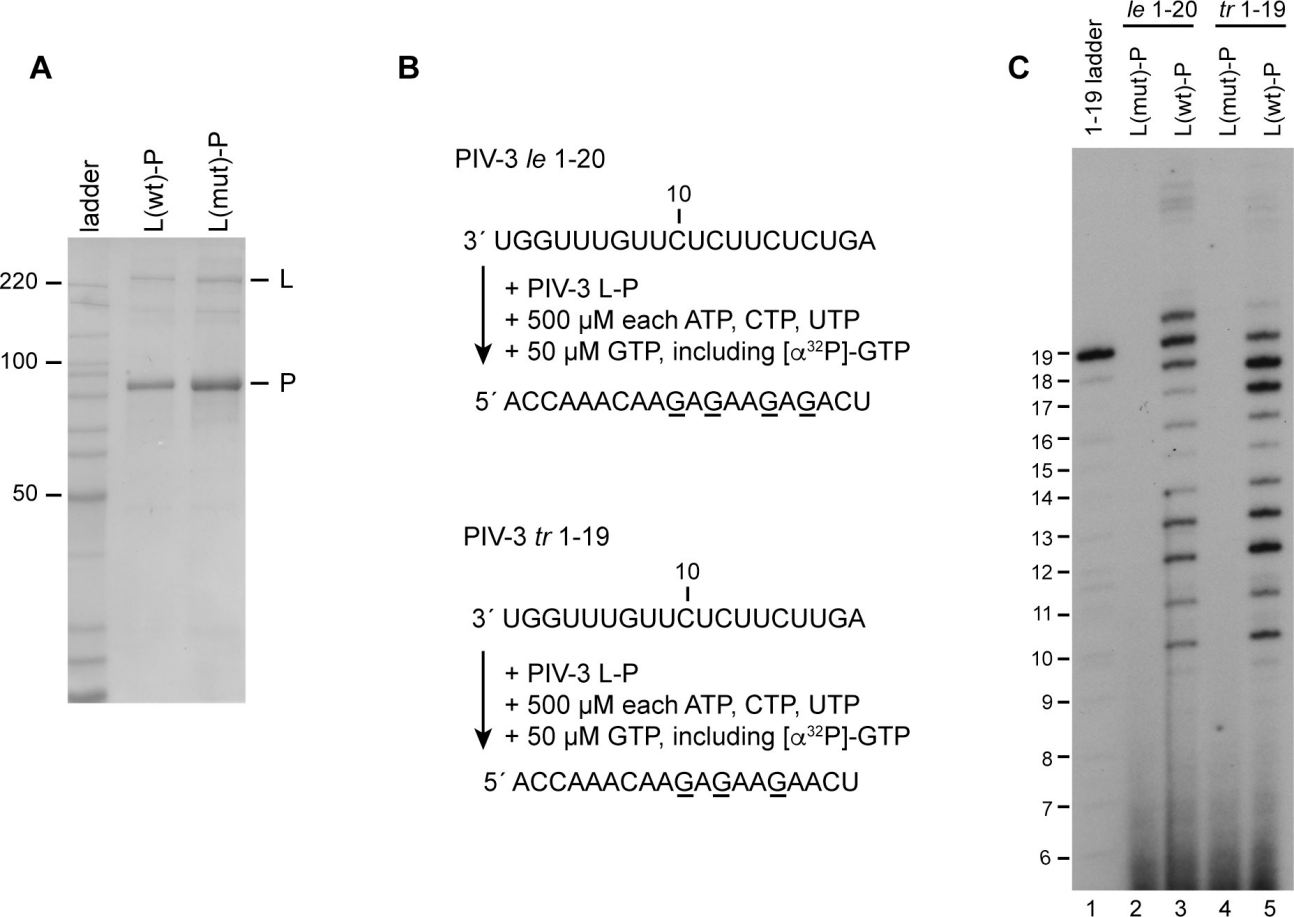
The PIV-3 polymerase initiates at position 1U of the PIV-3 *le* and *tr* promoters. (A) SDS-PAGE analysis of wt and variant PIV-3 L-P preparations detected by colloidal Coomassie blue staining. The variant L protein contained a D-to-A substitution in the GDN motif of the RNA polymerization domain. The first lane contains a Benchmark ladder. (B) Schematic diagram illustrating the PIV-3 *le* 1–20 and *tr* 1–19 templates, the NTP concentrations used, and the full-length products that would be generated following initiation at position 1U of each template. The underlined residues indicate the sites of radiolabel incorporation. (C) RNA synthesis products generated by the wt and variant polymerase, analyzed by denaturing polyacrylamide gel electrophoresis. The 1–19 ladder was generated by alkali hydrolysis of an ^32^P end-labeled RNA representing the complementary full-length product generated from position 1U of the PIV-3 *tr* 1–19 template.

We next assessed the initiation properties of the PIV-3 polymerase using a similar experimental design (Fig 2). The purified PIV-3 L-P complex (Fig 2A) was incubated with either a PIV-3 *le* 1–20 or *tr* 1–19 RNA template and NTPs. Because the PIV-3 polymerase was inefficient at incorporating a low concentration radiolabeled nucleotide opposite the 5´ end of the template, we used [α-^32^P]-GTP as the radioactive tracer together with 500 μM each of ATP, CTP and UTP (a low concentration of 50 μM unlabeled GTP was also included in the reactions to facilitate elongation). The first GTP incorporation site on both PIV-3 promoters is opposite position 10C (Fig 2B). Therefore, if the polymerase initiated at position 1, the smallest detectable product would be 10 nt; if it initiated internally, we would expect smaller products to be detected. On the *le* 1–20 template, the PIV-3 polymerase generated readily detectable products of 10 to 20 nt in length and on the *tr* 1–19 template, it generated products of 10 nt to 19 nt in length (Fig 2C, lanes 3 and 5, respectively). These results indicate that in contrast to the RSV polymerase, the PIV-3 polymerase initiated primarily at the 1U position.

### The PIV-3 polymerase can recognize the RSV promoter, but does not engage in back-priming

**Fig 3:**
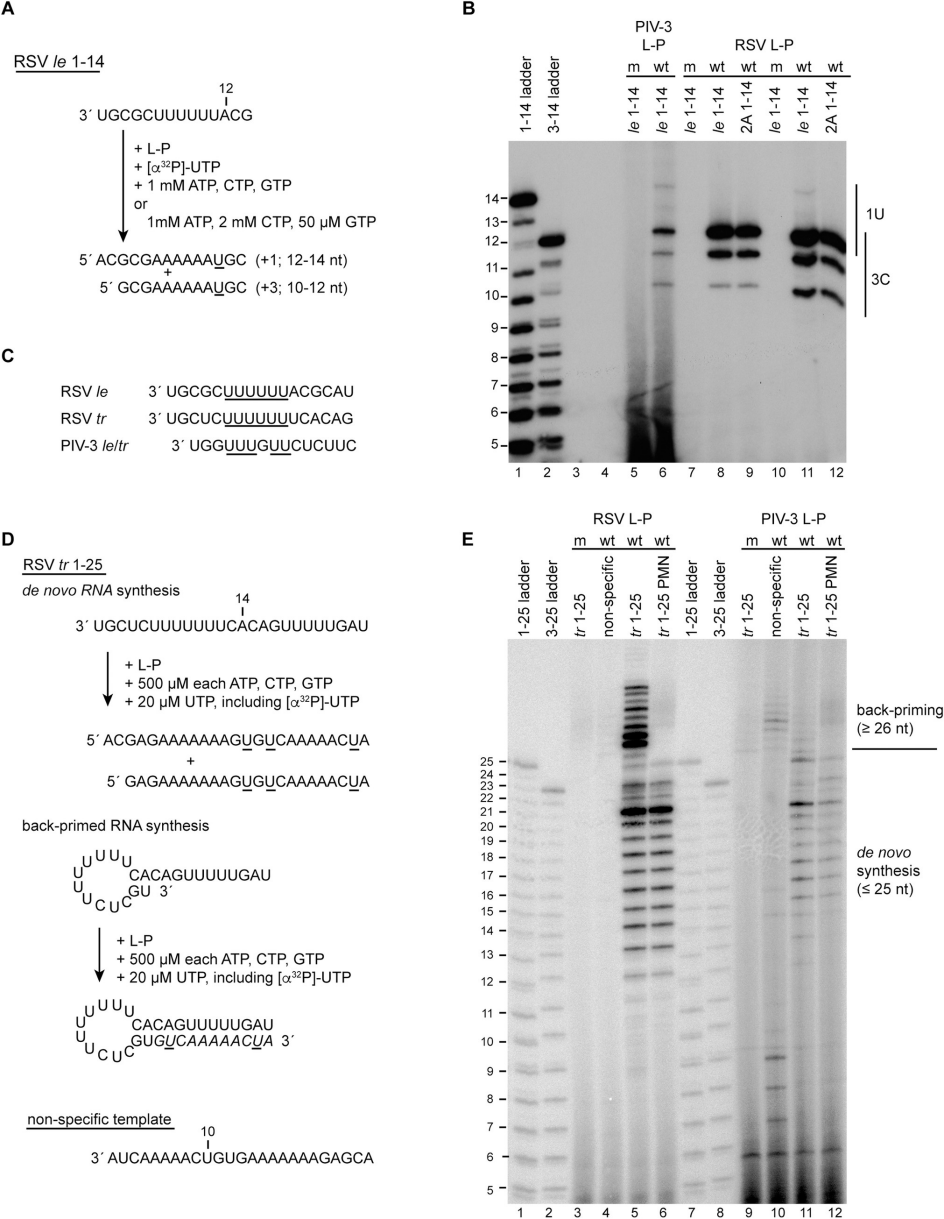
The PIV-3 polymerase initiates at positions 1U and 3C of the RSV promoter. (A) Schematic diagram illustrating the RSV *le* 1–14 template, the NTP concentrations used, and full-length products generated following initiation at 1U and 3C. The underlined residues indicate the sites of radiolabel incorporation. (B) RNA synthesis products generated by the wt and variant PIV-3 and RSV polymerases, analyzed by denaturing polyacrylamide gel electrophoresis. Lanes 9 and 12 show products generated from a template with a 2G-to-A substitution. Lanes 5–9 show products generated in the presence of equimolar ATP, CTP and GTP; lanes 10–12 show products generated under conditions with high CTP and low GTP concentrations. The 1–14 and 3–14 ladders represent the complementary products generated from position 1U and 3C of an RSV *le* 1–14 template. (C) Alignment of the RSV *le*, RSV *tr* and PIV-3 *le* and *tr* promoter sequences, with the U tract that is common to the promoters underlined. (D) Schematic diagram illustrating the RSV *tr* 1–25 template, the NTP concentrations that were used in the reactions, and the two RNA synthesis reactions that can occur from the RSV *tr* 1–25 promoter. In *de novo* RNA synthesis, RNA is generated by polymerase initiating at the promoter. In back-primed RNA synthesis, the *tr* RNA template folds into a secondary structure, with nt 1U and 2G base-pairing with nt 13C and 14A, and the 3´ end of the RNA is elongated in a templated fashion (as indicated with italicized residues). Sites of radiolabel incorporation in the *de novo* and back-primed RNA synthesis products are underlined. The sequence of the non-specific template is also shown. (E) RNA synthesis products generated by the wt and variant RSV and PIV-3 polymerases, analyzed by denaturing polyacrylamide gel electrophoresis. The 1–25 and 3–25 ladders represent the complementary products generated from positions 1U and 3C of an RSV *tr* 1–25 template. Lanes 6 and 12 show RNA generated from an RSV *tr* 1–25 template containing a 3´ puromycin (PMN) rather than hydroxyl group.

Previous studies have shown that RSV and PIV-3 polymerases have a stringent promoter sequence requirements [14,15,42]. However, although they are distinct, the RSV and PIV-3 promoters have sequence similarities: they begin with 3´ UG and include a pyrimidine-rich stretch. This suggested that the polymerase-promoter interactions might be sufficiently similar that the RSV and PIV-3 polymerases could recognize each other’s promoters. To test this possibility, we examined if the PIV-3 polymerase could initiate RNA synthesis from the RSV promoters. First, we tested its activity on the RSV *le* 1–14 nt template (Fig 3A). Similar to the data shown in Fig 1, almost all the detectable products of the RSV polymerase are initiated at position 3, with 1U-initiated RNA being barely detectable under equimolar ATP, CTP, and GTP conditions (Fig 3B, lane 8), and only a small amount of 1U-initiated RNA detectable under NTP concentrations biased to favor position 1 initiation (Fig 3B, lane 11; note that the gel in Fig 3B is less exposed than the gel in Fig 1C and so the 14 nt band generated under unbiased conditions is more difficult to detect). The RNAs initiated at position 3 could be clearly distinguished from those initiated at position 1 by comparison to products generated from a *le* promoter template bearing substitution at position 2, which prevents the RSV polymerase from initiating RNA synthesis from position 1 (Fig 3B, lanes 9 and 12). Likewise, the PIV-3 polymerase synthesized products of 10 to 14 nt in length from the RSV *le* promoter template, consistent with it also initiating at positions 1U and 3C (Fig 3B, lane 6). Although the PIV-3 polymerase utilized the same initiation sites as the RSV polymerase, the relative abundances of the products that it generated were different. The PIV-3 polymerase clearly generated a 14 nt product (indicative of initiation at position 1) under unbiased NTP conditions, and initiation at position 3C was relatively less dominant (compared to 1U initiation) than for RSV polymerase (Fig 3B, lanes 6 and 9). These findings show that the PIV-3 polymerase can recognize the RSV promoter, and that this promoter directs the polymerase to initiate at positions 1U and 3C. The ability of the PIV-3 polymerase to initiate at 3C was surprising, but could potentially be explained by alignment of the RSV and PIV-3 promoter sequences. As shown in Fig 3C, the U tract, which is common to the RSV and PIV-3 promoter sequences, is in alignment when position 1 of the PIV-3 promoters is aligned to position 3 of the RSV promoters. If this U-tract is part of the polymerase binding site, it could align the active site opposite position 3C of the RSV promoter. Thus, the PIV-3 polymerase could initiate at position 3C, similarly to the RSV polymerase, but has properties that favor initiation at position 1 to a greater extent than the RSV polymerase.

These data indicate that the PIV-3 polymerase has sufficient flexibility to initiate at an internal site. We then tested if this correlated with an ability to engage in a back-priming activity. To test this, we examined its behavior on an RSV *tr* 1–25 template, which serves as a substrate for an efficient back-priming reaction, resulting in products that are longer than the input template (Fig 3D). The RSV or PIV-3 L-P complex was incubated with RSV *tr* 1–25 template, and NTPs, including [α-^32^P]-UTP as a radioactive tracer. Under these conditions, the RSV polymerase generated a ladder of products 12–25 nt in length, consistent with initiation at positions 1U and 3C and elongation to the end of the template. The most prominent band amongst these products is at 21 nt, indicative of premature termination, consistent with previously published data [21]. In addition, products longer than 25 nt were readily detected, indicative of back-priming (Fig 3E, lane 5) [21]. These longer products were not detected if the template contained a puromycin group at the 3´ end, confirming that they resulted from nucleotide addition to the 3´ end of the template (Fig 3E, lane 6). Analysis of the RNA produced by the PIV-3 polymerase showed that it generated products up to 25 nt in length, but only very low levels of products longer than 25 nt, regardless of whether the template had a 3´ puromycin block (Fig 3E, lanes 11 and 12). The fact that the products generated by the PIV-3 polymerase from the RSV *tr* promoter migrated similarly to the 12–25 nt products synthesized by the RSV polymerase is consistent with the PIV-3 polymerase having initiated at positions 1U and 3C of the RSV promoter, as shown in Fig 3B. However, the fact that products longer than 25 nt were only barely detected indicates that the PIV-3 polymerase did not readily engage in back-priming, even if provided a template with the potential of adopting a back-priming structure. In contrast to the RSV polymerase, the PIV-3 polymerase generated some products from a non-specific RNA template (Fig 3E, compare lanes 4 and 10), which could represent RNAs initiated at an internal UG motif at positions 10 and 11 or 12 and 13 (Fig 3D), which could suggest that it is somewhat promiscuous in initiation site selection. On this template, it also generated some longer products that could be due to back-priming, but these were relatively faint. Together, these data confirm that the PIV-3 polymerase is capable of recognizing the RSV promoter to initiate *de novo* RNA synthesis but show that it does not use it as efficiently as the RSV polymerase as a substrate for back-priming activity.

### The RSV polymerase can recognize the PIV-3 promoter

**Fig 4:**
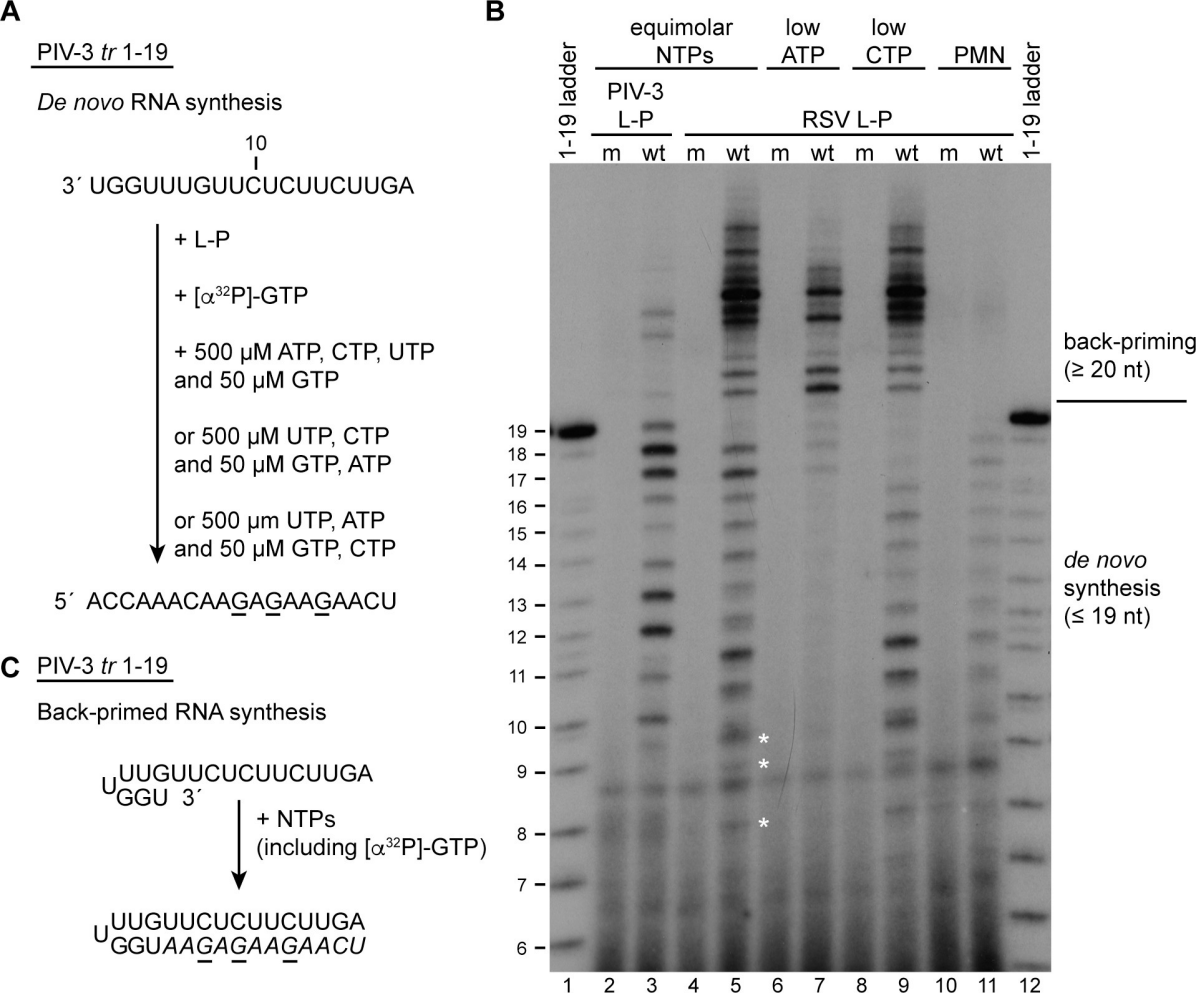
The RSV polymerase can utilize the PIV-3 promoter, but initiates at multiple sites. Schematic diagram illustrating the PIV-3 *tr* 1–19 template, the NTP concentrations used, and the full-length product that would be generated following initiation at position 1U. The underlined residues indicate the sites of radiolabel incorporation. (B) RNA synthesis products generated by the wt and variant RSV and PIV-3 polymerases, analyzed by denaturing polyacrylamide gel electrophoresis. The 1–19 ladder represents the complementary products generated from position 1U of the PIV-3 *tr* 1–19 template. Lanes 10 and 11 show RNA generated from a PIV-3 *tr* 1–19 template containing a 3´ puromycin (PMN) group. (C) Schematic diagram illustrating the proposed back-priming reaction in which the PIV-3 *tr* 1–19 template forms a hairpin structure, and its 3´ end is elongated in a templated fashion to yield products ≤ 31 nt in length. The italicized nucleotides represent those added during extension of the back-primed RNA, which would include radiolabeled G residues (underlined).

We then tested if the RSV polymerase could recognize the PIV-3 *tr* 1–19 promoter (Fig 4). In a reaction containing [α-^32^P]-GTP as the radioactive tracer, the PIV-3 polymerase generated a ladder of radiolabeled products from 10 to 19 nt in length, consistent with initiation from position 1 of the template, as shown in Fig 2, and some faint bands representing products >20 nt in length (Fig 4B, lane 3). Using this PIV-3 promoter template, the RSV polymerase also synthesized products up to 19 nt in length, although the band representing full length product was very weak. Products longer than 19 nt were also generated and were much more dominant than in the case of the PIV-3 polymerase (Fig 4B, compare lanes 3 and 5). Given the propensity of the RSV polymerase to engage in back-priming, we visually examined the PIV-3 promoter sequence to determine if it has the potential to form a hairpin structure that could be used as a substrate for back-priming. As shown in Fig 4C, the potential for a hairpin structure exists, specifically, nucleotides 1U, 2G, 3G have the potential to base-pair with nucleotides 5U, 6U and 7G. Subsequent elongation of the 3´ end of this RNA would result in radiolabeled products ranging from 22 to 31 nt in length (Fig 4C). Previous studies with the RSV polymerase have shown that it can also perform back-priming with hairpin mismatches [21]. The products > 19 nt produced by the RSV polymerase from the PIV-3 promoter ranged from 21 to ~ 30 nt in length. It seems likely that the ≥ 22 nt products were generated by back-priming activity according to the model in Fig 4C, and that the 21 nt radiolabeled RNA was either generated by back-priming from a hairpin with a mismatch or by a terminal transferase activity. To confirm that the products longer than 19 nt were generated by elongation of the 3´ end of the template, we tested a template sequence containing a 3´ puromycin group. In this case, the RSV polymerase yielded products up to 19 nt in length, but no longer products (Fig 4B, lane 11). Thus, the RSV polymerase was capable of initiating RNA synthesis on the PIV-3 promoter and efficiently using it as a substrate for back-priming.

Close inspection of the products generated by the PIV-3 and RSV polymerases revealed that whereas the smallest product that could be detected above background generated by the PIV-3 polymerase was 10 nt, consistent with initiation at position 1, the RSV polymerase generated products smaller than 10 nt (indicated with asterisks), and several products between 10 and 15 nt in length migrated as doublets. This suggested that the RSV polymerase was capable of initiation at one or more internal sites, in addition to position 1, yielding a mixture of RNA products with slightly different migration properties (Fig 4B, compare lanes 3 and 5). We reasoned that, given that there are multiple U residues near the 3´ end of the PIV-3 promoter, the RSV polymerase might be using an internal U residue as an initiation site, in addition to the 3´ terminal U residue. To test if this was the case, we investigated the effect of reducing the concentrations of ATP and CTP. As described above, the RSV polymerase requires high concentrations of the initiating NTPs, ATP and CTP to initiate opposite the 3´ terminal UG of its own promoter. If the RSV polymerase initiated exclusively at position 1U of the template, we would expect that reduction of either ATP or CTP would inhibit *de novo* RNA synthesis; if it initiated at an internal U residue in addition to, or instead of at 1U, we would expect that reduction of ATP would inhibit *de novo* RNA synthesis from the 1U site and the internal U site, but reduction of CTP would only inhibit initiation from the 1U site. When the ATP concentration was decreased to 50 μM, *de novo* synthesis from the promoter was almost completely inhibited (Fig 4B, compare lanes 5 and 7), consistent with ATP being one of the initiating nucleotides. The faint 18 and 19 nt bands that could be detected are likely due to low level degradation of the abundant back-priming product. When the CTP concentration was decreased to 50 μM, the RSV polymerase synthesized RNA products but the longest readily detectable product (aside from the back-priming products) was 16 nt in length (Fig 4B, lane 9). This is unlikely to be due to premature termination because the only CTP incorporation site following incorporation of the radiolabeled GTP tracer is at position 18; if reducing CTP concentration were inhibiting elongation at this incorporation site, it would be expected that a 17 nt product would be generated. Thus, this result is consistent with the RSV polymerase initiating at position 4 (and possibly other internal sites) in addition to the 1U site. Taken together, these findings indicate that the RSV polymerase could utilize the PIV-3 promoter as a template, and that it had similar properties as on its own promoter, initiating at position 1 and an internal position and likely engaging in a back-priming activity.

### MARV polymerase initiates at position 1 of its own promoter, but from position 2 on the EBOV promoter

**Fig 5:**
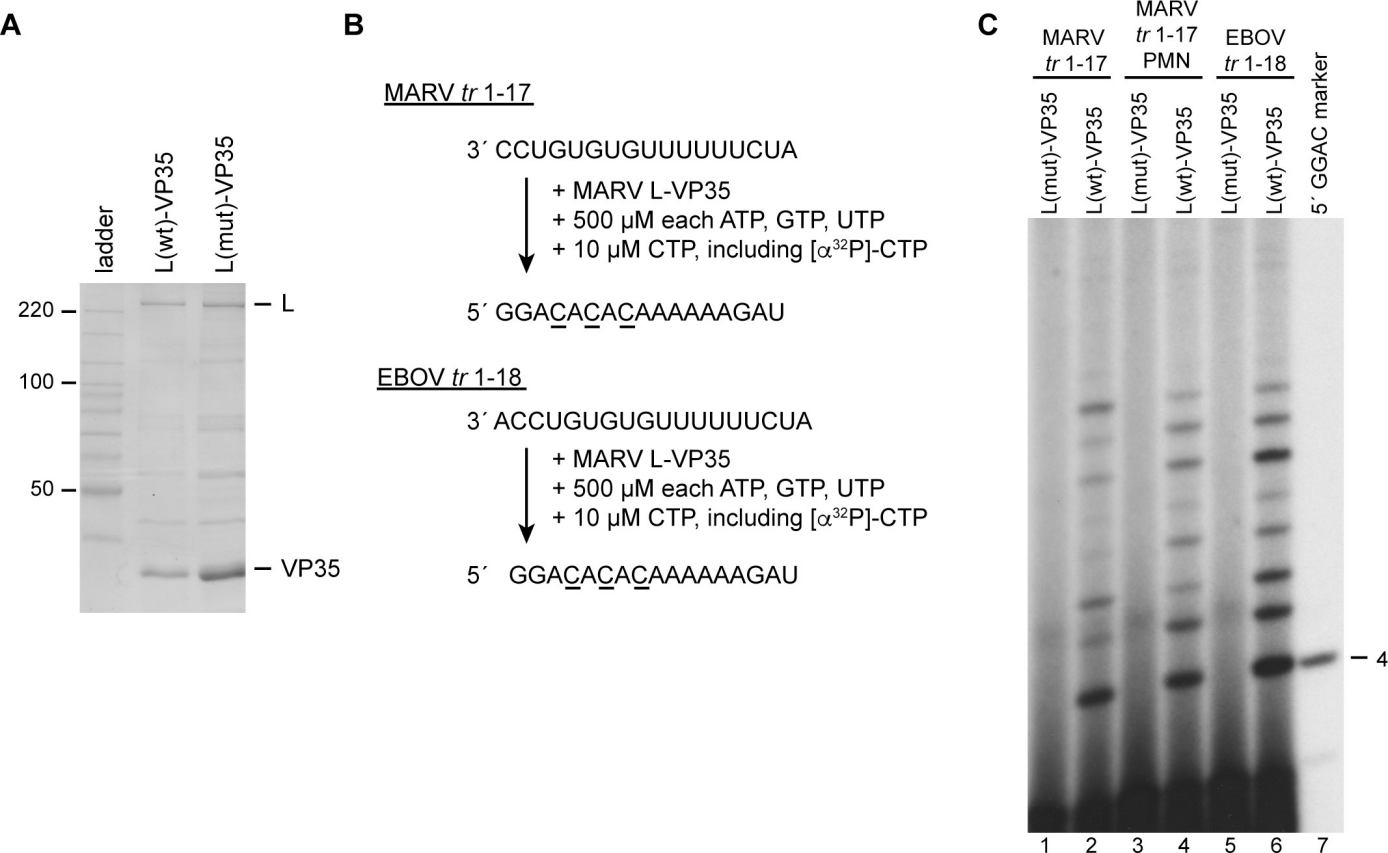
The MARV polymerase initiates at position 1 of its own promoter and at position 2 of the EBOV promoter. (A) SDS-PAGE analysis of wt and variant MARV L-VP35 preparations detected by colloidal Coomassie blue staining. The variant L protein contains a D-to-A substitution in the active site of the RNA polymerization domain. The first lane contains a Benchmark ladder. (B) Schematic diagram illustrating the MARV *tr* 1–17 and EBOV *tr* 1–18 templates, the NTP concentrations used, and the full-length products that would be generated following initiation at positions 1C or 2C, respectively. The underlined residues indicate the sites of radiolabel incorporation. (C) RNA synthesis products generated by the wt and variant polymerase, analyzed by denaturing polyacrylamide gel electrophoresis. Lanes 3 and 4 show RNA generated from a MARV *tr* 1–17 template containing a 3´ puromycin (PMN) group. The 5´ GGAC marker (lane 7) was generated using T7 RNA polymerase, such that it contains a 5´ triphosphate group to enable alignment with the four nucleotide RNA product.

We next examined the properties of the filovirus promoters using the MARV polymerase. A previous study of ebolavirus promoters had shown that within the same infection, the 3´ nucleotide of the promoter sequence can be variable, being either an A or G or absent [26]. Minigenome studies revealed that templates containing an additional 3´ nucleotide are viable and that the EBOV polymerase initiates at position 2, relative to the 3´ A or G residue (i.e. at the first C residue of the promoter). In contrast, MARV genome RNA lacks this additional 3´ nucleotide [43]. This raised the question of where does the MARV polymerase initiate on its own promoter, and where would it initiate on the EBOV promoter? To examine this, the L-VP35 complex of MARV was expressed and purified (Fig 5A) and incubated with a template representing either nucleotides 1–17 of the MARV *tr* promoter or 1–18 of the EBOV *tr* promoter, together with NTPs, including radiolabeled CTP (Fig 5B). The first incorporation site for CTP is at position 4 of the MARV *tr* 1–17 template and the radiolabeled products of the MARV polymerase ranged from 4–11 nt in length with some faint longer bands (Fig 5C, lanes 2 and 4). The identity of the 4 nt product was confirmed using a marker generated by T7 RNA polymerase (Fig 5C, lane 7). This result indicates that the MARV polymerase can initiate at position 1 of its own promoter. Analysis of the RNAs generated from the EBOV *tr* promoter showed that these were identical in length to those from the MARV promoter (Fig 5C, compare lanes 2 and 4 with lane 6). This showed that the MARV polymerase initiates at position 2 of the EBOV promoter, similarly to the EBOV polymerase. Thus, even though it initiates at the 3´ terminus of its own promoter, the MARV polymerase is readily capable of internal initiation.

## Discussion

It is generally assumed that most nsNSVs direct RNA synthesis initiation from the 3´ terminus of their genome and antigenome templates, and this might be true in most cases. However, studies are now revealing that different nsNSVs have evolved different RNA synthesis initiation patterns, with the EBOV polymerase being able to initiate with high efficiency at position 2, and the pneumovirus polymerases initiating with high efficiency at position 3 and less efficiently at position 1. Traditionally, initiation sites were determined by sequencing of the termini of viral genomes (e.g. following circularization of RNA isolated from viral particles) or primer extension analysis of viral RNA isolated from infected cells. While analysis of RNA isolated from virus particles or infected cells lends authenticity, these approaches leave open the possibility that small and/or highly unstable RNA products might not be detected, meaning that alternative initiation sites could be missed. The goal of this study was to use RNA oligonucleotide templates, representing the region of the RNA template exposed following N or NP dissociation for initiation complex formation, to characterize the initiation properties of the polymerases of PIV-3, a paramyxovirus, and MARV, a filovirus, using an *in vitro* RNA synthesis assay to capture small and unstable products. Specifically, we asked if a paramyxovirus and a filovirus polymerase utilized a single or multiple initiation sites, and if there are fundamental differences between these and the RSV polymerase to facilitate different initiation strategies. We also examined back-priming activity in an attempt to gain more information regarding this enigmatic process.

The data presented shows that whereas the RSV polymerase initiates at positions 1 and 3 of its promoters (Fig 1), PIV-3 and MARV polymerases initiate exclusively at position 1 of their own promoters (Figs 2 and 5). In the case of RSV, previous studies have found evidence that the two initiation sites dictate different outcomes, with initiation at position 1 beginning the process of replication and initiation at position 3 beginning the process of mRNA transcription [22,23,44]. In the case of PIV-3 and MARV, it is possible that position 1 is the initiation site for both transcription and RNA replication. Similarly, although the EBOV polymerase initiates at position 2 rather than position 1, it is possible that position 2 is its transcription and replication initiation site. The evolution of promoters with a single initiation site versus two initiation sites could be significant for understanding the mechanisms by which the balance of transcription versus replication is regulated during infection. A long-standing model of nsNSV transcription and replication is that the polymerase initiates at position 1 of the *le* promoter region for both processes [45–47]. According to this model, commitment to transcription versus replication occurs depending on whether the polymerase releases the positive sense le transcript and reinitiates at the first *gene start* signal to begin transcription, or continues to elongate the nascent RNA, to begin replication. This “decision” could be determined by N protein concentration and/or other factors. In contrast, in the pneumoviruses, where there are two initiation sites, the commitment to transcription versus replication can occur prior to the polymerase beginning RNA synthesis and does not appear to be governed by N protein concentration [25,48].

The fact that the PIV-3 and MARV polymerases initiate exclusively at position 1 of their own promoters, whereas the RSV and EBOV polymerases can initiate at internal sites, raised the possibility that these different polymerases might possess different structural features to facilitate initiation. Precedence for this comes from studies of the influenza virus polymerase, which adopts different conformations for replication initiation from the 3´ terminus of the vRNA promoter versus initiation at an internal site on the cRNA promoter [40,41,49]. Specifically, polymerases that initiate RNA synthesis at the 3´ end of the template by a *de novo* (primer independent) initiation mechanism typically have a priming loop and priming residue to stabilize the initiation complex and to help position the 3´ end of the template appropriately. We envisaged that polymerases that can initiate internally might either have a more flexible priming loop positioning or, in the case of the pneumoviruses, might have evolved a different mechanism to enable *de novo* initiation from position 3. If so, these features would not necessarily be shared with polymerases that initiate exclusively at position 1 of their own promoters. Apo structures are now available for the polymerases of VSV, rabies, RSV, HMPV, and parainfluenza virus type 5 (PIV-5) [7–13] but at present it is not possible to relate these structures to the data presented here because the structural features required for RNA synthesis initiation are poorly defined for the pneumoviruses, paramyxoviruses and filoviruses. The VSV and rabies virus polymerases have a priming loop that extends into the RNA dependent RNA polymerase active site, positioning a priming residue appropriately for *de novo* initiation [10–12,35]. In the other polymerase structures, this loop is retracted and is more integrated with the capping domain in which it also plays a functional role [7–9,13]. The PIV-5 polymerase structure shows instead an intrusion loop that extends into the RNA dependent RNA polymerase active site (although it is not positioned identically to the rhabdovirus priming loop) [7], but this too is retracted in the RSV and HMPV structures [8,9,13]. The priming loop that was identified in VSV and rabies virus polymerases is poorly conserved in primary amino acid sequence between nsNSVs and there is no clearly conserved priming residue [13]. Furthermore, studies with RSV failed to identify a priming residue in the same position as that of VSV and rabies virus [13]. Therefore, it is not clear where the priming loop and residue are for the paramyxoviruses or pneumoviruses (or filoviruses). Despite the difference in positioning of the intrusion loop between the paramyxovirus and pneumovirus polymerases, the data presented here show that the PIV-3 polymerase is not constrained to initiate only at position 1 and can initiate efficiently from an internal site (Fig 3). Likewise, the MARV polymerase can initiate internally on the EBOV promoter (Fig 5). Similarly, the polymerase of the paramyxovirus Sendai virus can initiate internally if additional nucleotides are added to the 3´ end of its promoter [50]. In addition, although the VSV polymerase appears to be highly constrained to initiate at the 3´ terminus of its promoter, the rabies virus polymerase initiates both at the 3´ end and at an internal site [17,27,35]. Thus, VSV polymerase appears to be exceptional amongst polymerases that have been examined to date in that it cannot initiate efficiently from an internal site, whereas the other polymerases appear to be flexible, regardless of their apo structure conformation.

The results showed that the promoter sequences could play a key role in determining initiation site. The MARV and EBOV promoter sequences are almost identical with each other, with position 1 of the MARV promoter clearly aligning with position 2 of the EBOV promoter sequence (in the case where the EBOV promoter has an additional 3´ nucleotide). Consistent with this, the MARV polymerase initiates very efficiently at position 2 of the EBOV promoter. More remarkably, the PIV-3 polymerase initiated at positions 1 and 3 of the RSV promoter, like the RSV polymerase, with initiation at position 3 being dominant. The preference for PIV-3 initiation at position 3 could be explained by alignment of the RSV and PIV-3 promoter sequences, which revealed that the U stretch, which is common to both the RSV and PIV-3 promoters, is in alignment if nucleotide 1 of the PIV-3 promoter is aligned to position 3C of the RSV promoter (Fig 3C). It is possible that this U stretch is a polymerase binding site and that when the PIV-3 polymerase binds to the RSV promoter, this is its preferred site positioning the active site at nucleotide 3C. Although the U-tract appears to play a key role, when the PIV-3 polymerase was provided a non-specific RNA template in which a U tract was not present, it initiated from an internal site, possibly from a UG motif that mimics positions 1 and 2 of its own promoter (Fig 3E). Thus, evidence indicates that many nsNSV polymerases are flexible in their interactions with the promoter, enabling them to initiate either at the 3´ terminus or at an internal site, with promoter sequence elements playing an important role in dictating initiation site selection. Although the polymerases appear to be sufficiently flexible to tolerate internal initiation sites, and the promoter sequences clearly play a key role in determining what site will be utilized, the RSV and PIV-3 polymerases had distinctive preferences for internal versus 3´ terminal initiation. For example, although the PIV-3 polymerase initiated at the same sites as the RSV polymerase on the RSV *le* promoter, the two polymerases differed in their efficiency at initiating at position 1U, relative to position 3C (Fig 3). Likewise, whereas the PIV-3 polymerase appeared to initiate exclusively at position 1U of its own promoter, the RSV polymerase initiated at internal sites on this promoter (Fig 4). A caveat to the interpretation of these results is that it is possible that the PIV-3 polymerase was highly efficient at initiating at internal sites, even on its own promoter, but could not elongate internally initiated products very efficiently. If this were the case, these internally initiated products would not be detectable if they had not incorporated the radiolabeled nucleotide. While this caveat cannot be excluded, it seems likely that the RSV and PIV-3 polymerases do have inherent properties that help to determine their initiation sites.

Another property of the pneumovirus polymerases is their propensity to use the template RNA to engage in a back-priming reaction [13,21,51]. Analysis of the PIV-3 polymerase on either its own or the RSV template revealed that it had little back-priming activity, as compared to the RSV polymerase (Figs 3 and 4). One caveat to this experiment is that the PIV-3 polymerase was examined in on-bead assays and it is possible that it was more constrained than the RSV polymerase. However, the PIV-3 polymerase was retained on beads via a tag at the C-terminus of P, which would normally be tethered to the N-RNA template, and it was clearly capable of performing *de novo* initiation and elongation, indicating that L could adopt different conformations. Therefore, we think that it is unlikely that this caveat would have affected these experimental results. It should be noted that back-priming (on an RSV template) has been reported for the polymerase of measles virus [52], which suggests that there might be differences between different paramyxoviruses. The role of back-priming in RSV infection is not known, but the dominance of this activity suggests that it might play an important function. It is possible that the conformation adopted by the apo forms of the RSV and HMPV polymerases, with the priming and intrusion loops folded away from the RNA dependent RNA polymerase active site, creates space for the back-priming template structure [8,9,13].

In conclusion, comparison of three different nsNSV families reveals that although they have evolved to have different RNA synthesis initiation strategies, their polymerases are remarkably flexible, with the capability of both 3´ terminal and internal initiation and the promoter sequences playing a key role in determining what site(s) are used. However, the polymerases do differ in their ability to engage in back-priming activity, at least under the conditions tested, suggesting that this might reflect a fundamental difference in the structures that different nsNSV polymerases can adopt.

## Materials and methods

### Polymerase complex expression plasmid construction

The RSV and PIV-3 L-P and MARV L-VP35 complexes were expressed using baculovirus vectors. To construct the recombinant baculoviruses, the viral L and P or VP35 open reading frame (ORF) sequences were inserted into pFastbac Dual vector (Invitrogen), with the L ORF being inserted under the control of the polyhedrin promoter and the P or VP35 ORF being under the control of the P10 promoter. The RSV L-P pFastbac dual construct has been described previously [21]. Briefly, the L and P coding sequences are derived from human respiratory syncytial virus strain A2 (Genbank accession number KT992094.1). The L ORF was codon optimized and chemically synthesized (GeneArt) whereas the P ORF was native sequence. A terminal tobacco etch virus (TEV) protease cleavage site and hexahistidine tag were inserted at the C-terminus of the P ORF. The PIV3 L and P coding sequences were derived from human parainfluenza virus type 3 strain JS (GenBank accession number NC_001796.2). Codon optimized and chemically synthesized versions of L and P (DNA 2.0) were inserted with a FLAG tag at the N terminus of L and an octahistidine tag on the C-terminus of P. The MARV L and VP35 coding sequences were derived from Lake Victoria marburgvirus strain Musoke (GenBank accession number DQ217792.1) and were codon optimized and chemically synthesized (DNA 2.0). An HA tag and TEV protease cleavage site were inserted at the N-terminus of L, and a TEV cleavage site and hexahistidine tag were inserted at the C terminus of VP35. A mutated version of each plasmid was also generated in which the catalytic aspartic residue of the GDNQ motif in the RNA dependent RNA polymerase domain of each of the L proteins was substituted with alanine. The following substitutions were made (amino acid numbering is relative to the untagged protein sequence): RSV L D811A, PIV-3 L D773A, MARV L D745A. The protein variants generated from these clones are referred to as L(mut)-P (in the case of RSV and PIV-3) or L(mut)-VP35 (in the case of MARV).

### Purification of RSV and HPIV3 L-P and MARV L-VP35 complexes

Recombinant baculoviruses expressing the L and P or L and VP35 proteins were generated using the Bac-to-Bac system (Invitrogen). The proteins were expressed in Sf21 insect cells. Cell pellets were collected and lysed in lysis buffer containing 50 mM NaH_2_PO_4_ (Sigma), 150 mM NaCl (Sigma), 0.5% NP40 (IGEPAL CA-630, Sigma), pH 8.0, protease inhibitors (cOmplete Mini EDTA-free Protease Inhibitor Cocktail, Roche) and 20 mM imidazole. RSV L-P complexes were extracted from cell lysates using affinity chromatography with Ni-NTA agarose resin (Invitrogen). L-P complexes bound to beads were washed three times with lysis buffer containing 60 mM imidazole and once with lysis buffer containing 100 mM imidazole. L-P complexes were eluted from the agarose resin with lysis buffer containing 250 mM imidazole. The eluted protein complexes were dialyzed into a buffer containing 150 mM NaCl, 20 mM Tris pH 7.4, 10% glycerol. PIV3 L-P and MARV L-VP35 complexes were extracted from cell lysates using affinity chromatography with His-tag Isolation and Pulldown Dynabeads (Invitrogen) using the same lysis and wash buffers as described above, except that for purification of the PIV-3 L-P complex, beads were washed three times with 60 mM imidazole containing buffer, whereas for the MARV L-VP35 complex, beads were washed two times with 60 mM imidazole and two times with 100 mM imidazole containing buffer. The isolated HPIV-3 L-P and MARV L-VP35 protein complexes were retained on beads (by virtue of the histidine tags inserted at the C-terminus of P or VP35) in 150 mM NaCl, 20 mM Tris pH 7.4, 1 mM DTT, 10% glycerol buffer; while the PIV-3 polymerase could be isolated in enzymatically active, soluble form, we were able to obtain higher quality on-bead preparations; we been unable so far to identify conditions that allow release of soluble MARV L-VP35 complexes from beads, likely due to protein aggregation. All isolated polymerase complexes were analyzed by SDS- PAGE and PageBlue staining (Fermentas) and the L protein concentration was estimated by comparing its band intensity against bovine serum albumin reference standards.

### In vitro RNA synthesis assays

All reactions were performed with the following conditions: 2 μM RNA oligonucleotide (Dharmacon), 50 mM Tris, pH 7.4, 8 mM MgCl_2_, 5 mM DTT, 10% glycerol and varying ATP, CTP, GTP, UTP concentrations as indicated in the figure legends for each experiment. For the experiment shown in Fig 1, all NTPs (except the radiolabeled UTP tracer) were included at 1 mM each to promote initiation; for the experiments in the remaining figures, NTPs (except the NTP used as the tracer) were included at 500 μM each, a concentration that slightly increases RSV back-priming efficiency compared to 1 mM each NTP [51]. In the experiments shown in Figs 2–5, cold NTP corresponding to the radiolabeled NTP was included at a low concentration that was determined to enable polymerase elongation to the end of the template without outcompeting the radiolabeled NTP. Other NTP concentration variations to modulate initiation from different sites are described in the text. Reactions contained 10 μCi of a radiolabeled NTP as a tracer. Reaction mixes were incubated at 30°C for 5 minutes prior to addition of L-P or L-VP35 complexes at a concentration of ~8 nM L protein (note that reactions with PIV-3 L-P and MARV L-VP35 were performed as on-bead reactions). Reactions were then incubated at 30°C for 1 h, then heat inactivated at 90°C for 3 minutes and incubated on ice for 2 minutes. The RNA was extracted with acid phenol-chloroform and ethanol precipitation. RNA pellets were resuspended in RNase free water and stop buffer (deionized formamide, 10 mM EDTA, bromophenol blue, xylene cyanol). The purified RNA products were migrated alongside molecular weight ladders representative of the product size on either a 20% polyacrylamide gel containing 7 M urea in Tris-borate-EDTA buffer (for products ≥ 8 nt) or a 25% polyacrylamide gel containing 7 M urea in Tris-taurine-EDTA buffer (for products ≤ 7 nt). The 20% gels were vacuum dried onto 3MM paper at 80°C. All gels were analyzed using phosphorimaging or autoradiography.

### Generation of size markers

RNA products ≥ 10 nt were analyzed alongside size markers generated by alkali hydrolysis of an end-labeled RNA oligonucleotide of the same sequence as the anticipated full length product, as described previously [21]. 5΄ triphosphorylated RNA markers used to detect 4 nt products were generated using an adaption of a previously described method [53]. DNA oligonucleotides 5´- TAATACGACTCACTATA-3´ and 3´ ATTATGCTGAGTGATAT**CCTG** 5´ were annealed to create an oligo duplex of the Class II T7 promoter with a template overhang (in bold). Annealed oligonucleotides were prepared by mixing 4 μM short oligonucleotide and 4 μM long overhang (template) oligonucleotide in Tris-EDTA pH 8.0, incubated at 90°C for 3 minutes, then allowed to cool to room temperature. 40 μL *in vitro* transcription reactions were prepared with the following conditions: 40 mM Tris-HCl (pH 8.0), 20 mM MgCl_2_, 2 mM spermidine,10 mM DTT, 0.1 mg/mL bovine serum albumin, 2 mM ATP, 1 mM CTP, 1 mM GTP; 1 μCi [α-^32^P] CTP, 0.1 μM DNA annealed oligos and 1 unit/μL T7 RNA polymerase (New England Biolabs). Reactions were incubated at 37°C for 5 hours. Samples were treated with 1 μL DNase I (New England Biolabs) for 10 minutes at 37°C. Reactions were quenched by adding 1 μL of 0.5 M EDTA. Products were subjected to phenol chloroform extraction and ethanol precipitation prior to use as markers.
